# Prevalence of active foot disease and foot disease risk factors in a subacute inpatient rehabilitation facility: a cross-sectional prevalence study

**DOI:** 10.1186/s13047-014-0041-x

**Published:** 2014-10-09

**Authors:** Brenton J Earl, Peter A Lazzarini, Ewan M Kinnear, Petrea L Cornwell

**Affiliations:** Department of Podiatry, Metro North Hospital and Health Service, Queensland Health, Brisbane, Queensland 4032 Australia; School of Medicine, Flinders University, Adelaide, South Australia 5001 Australia; Allied Health Research Collaborative, Metro North Hospital and Health Service, Queensland Health, The Prince Charles Hospital, Rode Road, Chermside, Brisbane, Queensland 4032 Australia; School of Clinical Sciences, Queensland University of Technology, Brisbane, Queensland 4059 Australia; Griffith Health Institute, Behavioural Basis of Health, Griffith University, Brisbane, Queensland 4122 Australia

**Keywords:** Subacute, Inpatient, Foot, Complication, Prevalence

## Abstract

**Background:**

Australian subacute inpatient rehabilitation facilities face significant challenges from the ageing population and the increasing burden of chronic disease. Foot disease complications are a negative consequence of many chronic diseases. With the rapid expansion of subacute rehabilitation inpatient services, it seems imperative to investigate the prevalence of foot disease and foot disease risk factors in this population. The primary aim of this cross-sectional study was to determine the prevalence of active foot disease and foot disease risk factors in a subacute inpatient rehabilitation facility.

**Methods:**

Eligible participants were all adults admitted at least overnight into a large Australian subacute inpatient rehabilitation facility over two different four week periods. Consenting participants underwent a short non-invasive foot examination by a podiatrist utilising the validated Queensland Health High Risk Foot Form to collect data on age, sex, medical co-morbidity history, foot disease risk factor history and clinically diagnosed foot disease complications and foot disease risk factors. Descriptive statistics were used to determine the prevalence of clinically diagnosed foot disease complications, foot disease risk factors and groups of foot disease risk factors. Logistic regression analyses were used to investigate any associations between defined explanatory variables and appropriate foot disease outcome variables.

**Results:**

Overall, 85 (88%) of 97 people admitted to the facility during the study periods consented; mean age 80 (±9) years and 71% were female. The prevalence (95% confidence interval) of participants with active foot disease was 11.8% (6.3 – 20.5), 32.9% (23.9 – 43.5) had multiple foot disease risk factors, and overall, 56.5% (45.9 – 66.5) had at least one foot disease risk factor. A self-reported history of peripheral neuropathy diagnosis was independently associated with having multiple foot disease risk factors (OR 13.504, *p* = 0.001).

**Conclusion:**

This study highlights the potential significance of the burden of foot disease in subacute inpatient rehabilitation facilities. One in eight subacute inpatients were admitted with active foot disease and one in two with at least one foot disease risk factor in this study. It is recommended that further multi-site studies and management guidelines are required to address the foot disease burden in subacute inpatient rehabilitation facilities.

## Background

Subacute inpatient care plays a significant role in the Australian health care system, providing a valuable contribution to patient outcomes and is becoming increasingly essential for the flow of patients from acute care to community care [1]. There are over 53,000 admissions into subacute inpatient rehabilitation facilities in Australia each year [2]. The majority of admissions to a subacute inpatient rehabilitation facility occur after an acute inpatient admission for orthopaedic impairment (>35%), stroke (15%), brain injury, spinal injury, other neurological conditions and amputations (approximately 5% each) [2].

Numerous definitions surround the term subacute in the Australian health context. An emergent theme is that a subgroup of patients exists whose health care needs are no longer acute and directly influenced by their original principal medical diagnosis, but instead their health care needs are predicted by their functional status [1-6]. The most readily recognised type of subacute care is rehabilitation [1]. Rehabilitation commonly refers to medically directed multi-disciplinary services that aim to improve an individual’s function [3]. These services are based on an evidence-based comprehensive assessment of function and negotiated patient goals [1-3].

Admissions to Australian public subacute inpatient rehabilitation facilities are mostly for people aged over 70 years of age [2], with multiple chronic diseases [1] and often more disabled than those seen within private health facilities [2]. Chronic diseases are expected to be responsible for 80% of Australia’s overall disease burden by 2020 [7] and are said to be responsible for 87.6% of premature death and disability [8]. Furthermore chronic disease is very common in the older Australian population with 80% of those aged over 65 years reporting having three or more chronic diseases [9,10]. This significant potential increase in demand, from Australia’s older chronic disease-afflicted population, on the overall efficiency of the health system has been identified by government as a key focus area for action [11]. The “urgent need for substantial investment in, and expansion of, sub-acute services” to address this demand was recommended by the Australian National Reform Commission’s (2009) [12] to improve functional impairments in the hospitalised older person to avoid potentially poorer outcomes and higher acute re-admission rates [11,12].

Foot disease is generally the end result of chronic disease [13-15]. ‘Foot disease complications’, including foot ulcerations and foot infections, consume significant inpatient acute hospital resources in Australia [15-19]. They are the leading cause of amputations [15,16], a leading cause of diabetes-related hospitalisations [18,19] and have been reported to consume up to 5% of all hospital bed days in one study [17]. Amputations typically have a preceding foot disease complication that is the result of trauma and ‘foot disease risk factors’; including peripheral neuropathy, peripheral arterial disease (PAD) and orthopaedic foot deformity [15-21]. ‘At risk populations’ for foot disease are those populations more predisposed to developing foot disease risk factors and in turn foot disease complications that often progress to lower limb amputation [14]. Whilst people with diabetes are widely acknowledged as the primary at risk population for foot disease [15-19], there is growing evidence that other chronic disease patient groups, including those with cardiovascular disease (CVD) and chronic kidney disease (CKD), are also at risk populations with similar degrees of risk factors and foot disease to diabetes [20,21]. Furthermore studies suggest when some chronic diseases are combined foot disease rates can double [20,21].

Given the potential increased need for subacute inpatient rehabilitation services, and growth in the numbers of patients in at risk populations for foot disease in these services, it appears necessary to review the role foot disease and foot disease services may play in an expanded subacute inpatient rehabilitation sector. Currently there are limited studies that address the prevalence of foot disease and foot disease risk factors in subacute inpatient rehabilitation facilities [22,23]. As such one Australian guideline recommends podiatry and foot care staffing levels based entirely on expert opinion [24]. With national health reform recommendations urging the rapid expansion of these services it would appear an opportune time to investigate the prevalence of active foot disease and foot disease risk factors in subacute inpatient rehabilitation services.

The primary aim of this study was to determine the prevalence of active foot disease and foot disease risk factors in a subacute inpatient rehabilitation facility. The secondary aim was to determine any associations between demographic, medical co-morbidity history and foot disease history explanatory variables and foot disease outcome variables in a subacute inpatient rehabilitation facility.

## Methods

The setting for this cross-sectional study was a large public subacute inpatient rehabilitation facility in Queensland, Australia. At the time of this study the facility consisted of 66 beds across two wards. Ethical approval for this study was obtained from The Prince Charles Hospital Human Research Ethics Committee and informed consent was obtained from all individual participants for this study.

Participants included all consenting adults admitted for subacute inpatient rehabilitation care at the facility during two different four week periods; August – September 2011 and November – December 2011. Exclusion criteria included children, patients with a cognitive deficit, and those who did not provide informed written consent to participate in the study. Two distinct four week periods, rather than one longer period, were used by the authors in an attempt to reflect any seasonal variations in admissions in this population as per other Australian foot disease studies [25].

Consenting eligible participants underwent a short non-invasive foot examination by a podiatrist at a convenient time within the first 72 hours of their subacute inpatient rehabilitation facility admission. Each examination used the validated Queensland Health High Risk Foot Form (QHRFF) to collect age, sex, co-morbidity and foot disease data [14]. The QHRFF data collection procedures, methods and definitions have been previously reported [14]. In brief the QHRFF collects 46-items of data across seven broad domains via a survey of the patient’s medical history and a physical clinical assessment for foot disease complications and foot disease risk factors [14]. The seven domains include identifying general demographics, different health professionals attending (data not utilised for this study), medical co-morbidity history, foot disease risk factor history, clinical diagnoses of foot disease risk factors, clinical diagnoses of foot disease complications, and clinical management principles performed (data not utilised for this study) [14]. The domains of medical co-morbidity history and foot disease risk factor history were defined as the participant self-reporting being previously diagnosed by a health professional [14].

All foot disease complications and foot disease risk factors were clinically diagnosed by the podiatrist for the purposes of this study using definitions from the national diabetic foot guidelines and adopted by the validated QHRFF [14,19]. Foot ulceration was defined as a current full thickness wound beneath the ankle on a person with clinically diagnosed peripheral neuropathy or PAD [13,14]. Foot ulcer infection was defined by the presence of two or more clinical signs of infection in a current foot ulcer [13,14,19]. Acute Charcot joint was defined clinically as a red, hot swollen joint in a patient with peripheral neuropathy and no current foot ulceration [14,19]. Amputation was defined as the patient having a previous or current (defined as part of the most recent acute inpatient admission) amputation procedure of the lower limb [14]. Peripheral neuropathy was diagnosed by the absence of sensation to a 10 g monofilament on at least two plantar forefoot sites on the one foot [13,14,19]. PAD was the inability to palpate any pulses, ankle brachial indices < 0.9 or toe systolic pressures < 70 mmHg on at least one foot [13,14,19]. Foot deformity diagnosis required a score of three or more on the six foot deformity point scale on at least one foot [14,19].

The primary outcomes for the study were clinically diagnosed active foot disease complications or foot disease risk factors. Foot disease complications included current foot ulceration, foot ulceration infection, acute Charcot joint and amputation. Foot disease risk factors included previous foot ulceration, previous amputation, peripheral neuropathy, peripheral arterial disease (PAD) and foot deformity. Secondary outcome measures included grouping participants into those that align with existing foot risk groups, including multiple foot disease risk factors (high risk foot), single foot disease risk factor (at risk foot) and nil foot disease risk factors (low risk foot) [14,19].

### Statistical analysis

All data was analysed using SPSS 18.0 for Windows (SPSS Inc., Chicago, IL, USA) or GraphPad Software. Descriptive statistics were used to display the age, sex and medical co-morbidity history, foot disease risk factor history, clinical diagnosed foot disease complications and foot disease risk factors; using means and standard deviations (SD) for continuous variables or proportions (with 95% confidence intervals for outcome measures) for categorical variables. Chi-squared and ANOVA tests were used to test differences in explanatory variables between different outcome groups.

Univariate and multivariate logistic regression analyses were undertaken for outcome groups recording at least 20 cases to aid with the robustness of the analysis [26,27] and considered to require clinical management whilst in the subacute facility [14-19]. Variables that achieved a statistical significance of *p*< 0.25 at the univariate level were included in the initial multivariate logistic regression model [26]. A backwards stepwise method was used for the multivariate logistic regression, with non-significant (*p*> 0.05) variables removed at each step [26,27]. The Hosmer and Lemeshow Goodness of Fit and Omnibus tests were used to indicate the goodness of fit and significance of the model respectively [26,27]. A non-significant value (*p*> 0.05) for the Hosmer and Lemeshow test indicates the model is a good fit with the outcome variable and a significant value (*p*< 0.05) for the Omnibus test indicates the explanatory variables jointly are independently associated with the outcome variable [26,27].

## Results

Ninety seven patients were admitted to the subacute inpatient rehabilitation facility during the study period. Twelve patients were excluded due to a cognitive deficit or unwilling to consent to this study. Thus, 85 (88%) participants were included and examined for this study. The mean age of included participants was 80 (±9) years, age range 43 – 97 years and 60 (71%) were female. Table 1 displays the numbers and proportions of participants with the primary outcomes of clinically diagnosed active foot disease complications and foot disease risk factors. Overall, ten (11.8%) individual participants had one or more active foot disease complications; including ten with a current foot ulcer and eight with a current foot infection.

**Table 1:** **Number and proportions (%) of active foot disease complications and foot disease risk factors outcomes**

	**Number**	**% [95% CI]**
Active foot disease complications*		
Current foot ulcer	10	11.8 [6.3 – 20.5]
Current foot ulcer infection	8	9.4 [4.6 – 17.7]
Acute charcot	0	0 [NA]
Current amputation	1	1.2 [NA]
Foot disease risk factors*		
Previous foot ulcer	17	20.0 [12.8 – 29.8]
Previous amputation	0	0 [NA]
Peripheral neuropathy	21	24.7 [16.7 – 34.9]
Peripheral arterial disease	33	38.8 [29.2 – 49.5]
Foot deformity	31	36.5 [27.0 – 47.1]

*Note: Participants may have had more than one foot disease complications and/or foot disease risk factors. CI: Confidence Intervals; NA: Not appropriate as CI extends < 0.

Table 2 displays the medical co-morbidity history and foot disease risk factor history for all participants and the sub-groups of nil, single and multiple clinically diagnosed foot disease risk factors. Overall, 48 (56.5% (95% confidence intervals 45.9 – 66.5)) participants presented with at least one foot disease risk factor; including 20 (23.5% (15.7 – 33.6)) with a single foot disease risk factor and 28 (32.9% (23.9 – 43.5)) with multiple foot disease risk factors. The only significant differences between the sub-groups were that participants with foot disease risk factors were significantly more likely to have a self-reported history of the foot disease risk factors of peripheral neuropathy and peripheral arterial disease (PAD) (*p* < 0.01).

**Table 2:** **Numbers and proportions (%) of medical co-morbidity and foot disease history for foot disease subgroups**

	**All participants**		**Nil foot disease risk factors**		**Single foot disease risk factor**		**Multiple foot disease risk factors**		
	**Number**	**%**	**Number**	**%**	**Number**	**%**	**Number**	**%**	***p*** **value**
Co-morbidities	85	100	37	100	20	100	28	100	
Hypertension	52	61.2	22	59.5	15	75.0	15	53.6	0.311
Dyslipidaemia	41	48.2	17	45.9	14	70.0	10	35.7	0.060
Smoker	15	17.6	4	10.8	6	30.0	5	17.9	0.193
Diabetes	19	22.4	5	13.5	6	30.0	8	28.6	0.227
CVD	40	47.1	17	45.9	9	45.0	14	50.0	0.928
CKD	14	16.5	4	10.8	5	25.0	5	17.9	0.376
ESRF	4	4.7	0	0	1	5.0	3	10.7	NA
Neuropathy^#^	12	14.1	0	0	3	15.0	9	32.1	0.001
PAD^#^	15	17.6	0	0	6	30.0	9	32.1	0.001

^#^Self-reported history; CVD: Cardiovascular disease; CKD: Chronic kidney disease; ESRF: End stage renal failure; PAD: Peripheral arterial disease. NA: Not applicable to test as the assumption of Chi-squared test is violated as 2 cells had expected count < 5.

Table 3 displays the results of the univariate analysis for each of the explanatory variables against the outcome group of multiple clinically diagnosed foot disease risk factors. Unlike all other outcome groups, the multiple foot disease risk factor group was considered to be the most clinically relevant in regards to potentially requiring management during an inpatient stay [14,19], whilst, also having enough cases to allow more robust multivariate analyses to be performed [26,27]. Significant associations were found between the presence of multiple clinically diagnosed foot disease risk factors and a self-reported history of peripheral neuropathy and PAD (*p*< 0.05). The variables included in the subsequent multivariate analysis for multiple foot disease risk factors were dyslipidaemia, ESRF, self-reported PAD and self-reported peripheral neuropathy (*p*< 0.25). The final multivariate logistic regression model for the multiple foot disease risk factors group demonstrated that the explanatory variables of a self-reported history of peripheral neuropathy (OR 13.504 [2.857 – 63.818], *p* = 0.001) and dyslipidaemia (OR 0.281 (0.092 – 0.860) *p* = 0.026) were independently associated for this group (Hosmer and Lemeshow Goodness of Fit χ^2^ = 0.078, *p* = 0.780; Omnibus test χ^2^ = 16.107, *df* = 2, *p*< 0.001).

**Table 3:** **Univariate analysis of explanatory variables and multiple foot disease risk factor group outcome variable**

**Variable**	**Odds ratio (95% CI)**	***p*** **value**
Sex	1.214 (0.455 – 3.238)	0.700
Age	1.027 (0.974 – 1.083)	0.305
Hypertension	0.624 (0.248 – 1.566)	0.315
Dyslipidaemia	0.466 (0.183 – 1.184)	0.103*
Smoker	1.022 (0.313 – 3.338)	0.972
Diabetes	1.673 (0.585 – 4.786)	0.341
CVD	1.192 (0.482 – 2.95)	0.703
CKD	1.159 (0.349 – 3.853)	0.810
ESRF	6.720 (0.666 – 67.819)	0.077*
Neuropathy^#^	8.526 (2.087 – 34.831)	0.001**
PAD^#^	4.026 (1.263 – 12.838)	0.017**

* = *p* Value of < 0.25, resulting in variable being included into initial multivariate model.

** = *p* Value of < 0.05, indicating a correlation between variables.

^#^Self-reported history.

CI: Confidence Intervals; CVD: Cardiovascular disease; CKD: Chronic kidney disease; ESRF: End stage renal failure; PAD: Peripheral arterial disease.

## Discussion

This study is the first known to primarily investigate the prevalence of clinically diagnosed active foot disease complications and foot disease risk factors in patients admitted to a subacute inpatient rehabilitation facility. The results of this study indicate that nearly one in eight inpatients at this subacute inpatient facility had active foot disease placing them at high risk of lower limb amputation without urgent effective management. This risk of amputation was exacerbated by the very high proportion of clinical infection present in the active foot disease group. Furthermore one in every two inpatients had at least one foot disease risk factor placing them at risk of developing foot disease, whilst one in three inpatients had multiple foot disease risk factors. These outcomes highlight the potential burden of foot disease within the rapidly expanding Australian subacute inpatient sector and forecast the need to ensure evidence-based foot disease management is available in subacute inpatient rehabilitation facilities to prevent acute inpatient re-admission and amputation.

The prevalence of foot disease in this subacute inpatient population were similar, and in some cases higher, than those reported in other at risk populations for foot disease and other smaller subacute inpatient studies [15-23]. For example active foot disease prevalence reported in this study via current foot ulcers (12%) appears to broadly align with the foot ulcer prevalence reported in two other smaller subacute inpatient studies [22,23]; foot ulcer prevalence in those studies were 7% (Australian study) [22] and 15% (UK study) [23]. Furthermore, the current foot ulcer rate of this study compares with prevalence rates in other at risk populations; such as diabetes populations (4-10%) [28], people with diabetes receiving dialysis (12%) [29] and those with a co-diagnosis of diabetes and CKD (16%) [20]. The rate of clinical infection in the small number of foot ulcers in this study was very high (80%), however, interestingly this rate was similar to that of another large multi-site study reporting infection of those hospitalised with diabetic foot ulcers (82%) [13]. Conversely the current and previous amputation rate found in this study (1%) was much lower than those reported in other similar Australian subacute studies (5 – 7%) [2,22]. The authors hypothesise this may have either been a chance anomaly of the study periods chosen or that the implementation of best practice management occurring in Queensland at the time may have had some impact on the lower amputation rates [30,31].

In addition to those with active foot disease, a further 21% of participants in this study were classified as presenting with multiple foot disease risk factors placing them at high risk of developing foot disease if not managed effectively. This again aligns very closely with the 20% of the Australian diabetes population reported as having multiple foot disease risk factors [32]. The clinically diagnosed foot disease risk factor prevalence rates of peripheral neuropathy and PAD in this study were also found to be comparable or higher than other at risk populations. Our findings indicated that one in four (25%) subacute inpatient admissions had clinically diagnosed peripheral neuropathy which aligned with the aforementioned UK subacute inpatient study (26%) [23], and Australian studies of diabetes and CKD populations (~20%) [20,32]. The PAD prevalence found in this study population (39%) was much higher than the UK subacute inpatient study (11%) [23] and the Australian diabetes (16%) [32] and CKD (21%) [20] populations. This may be the result of studying an older frailer population with multiple chronic diseases and functional dependency that typically occur in subacute inpatient rehabilitation admissions. From a foot ulcer perspective, PAD is the most important foot disease risk factor that prevents foot ulcer healing [19,28,33] and is reported to be a contributing factor in 90% of diabetic amputations [33]. In the subacute setting PAD has also been associated with limited mobility, impaired functional status, falls and lower health-related quality of life [34,35]. Lastly, the foot deformity prevalence reported in this study (36%) was less than that reported in two other similar studies investigating foot deformity in inpatient populations (43 – 50%) [22,36]. It could be argued that this was understandable as the definition for determining an overall foot deformity score in this study was much more stringent (requiring three indicators of foot deformity [19]) than the single indicator of deformity used in other studies [22,36].

Peripheral neuropathy and PAD are commonly acknowledged as the leading foot disease risk factors for the development of foot disease [15-17] and this study was no different. Our findings suggest that a self-reported history of a previous health professional diagnosis of peripheral neuropathy independently increased the chance of having multiple foot disease risk factors. Interestingly, our findings suggest that having dyslipidaemia independently decreased the chance of having multiple foot disease risk factors. However, the authors postulate this may be the result of those diagnosed with dyslipidaemia having already implemented tight medication control over their lipid profile, and thus, decreasing the risk of developing foot disease risk factors [37].

The comparable prevalence of foot disease found in this study to those found in other similar at risk populations for foot disease suggests the need for comparable co-ordinated evidence-based approaches to foot disease management as occurs in other at risk populations. Studies consistently demonstrate evidence-based management of people with the foot disease complication of foot ulcers significantly reduces lower extremity amputation rates by up to 85% in at risk populations such as diabetes [19,29,33,38,39]. Evidence-based management of foot ulcers principally requires a coordinated multi-disciplinary team approach [19,29,30,38,39]. The Australian and international diabetic foot guidelines suggest a multi-disciplinary diabetic foot ulcer team should consist of at minimum a physician, podiatrist and nurse [19,40]. If the foot disease findings of this study are generalisable to other subacute inpatient facilities this highlights the potential importance of access to evidence-based multi-disciplinary foot teams within the subacute inpatient rehabilitation sector to prevent deterioration of active foot disease, re-admission into acute inpatient facilities and amputation.

The prevalence of foot disease risk factors found in this study is also comparable to other at risk populations for foot disease. Again Australian and international diabetes guidelines’ recommendations for the management of people with foot disease risk factors suggest routine podiatry review in a foot protection program to prevent foot disease [19]. The identification that over 50% of all participants admitted into this subacute inpatient rehabilitation facility had at least one foot disease risk factor adds weight to a smaller Australian subacute inpatient study that reported 41% of subacute inpatient participants had foot pathology requiring podiatric management [22]. Thus, it could be suggested that about half of patients admitted to subacute inpatient facilities may require podiatric management during or after their admission.

Furthermore diabetes guidelines recommend all people with diabetes should be assessed annually to determine their risk of developing foot disease [19]. These guidelines highlight the importance of screening and early identification of foot disease risk factors to monitor and prevent foot disease complications in the future [19]. This may also be required in the subacute population if the results of this study are generalisable. However, the findings of this study also suggest merely asking patients if they have been previously diagnosed with peripheral neuropathy may be a simpler way to identify those that may also have multiple foot disease risk factors. This may be an efficient strategy to triage those most likely to have foot disease risk factors for further assessment in resource constrained facilities rather than physically screening all admissions for foot disease.

The results of this study should be viewed in the context of several limitations. First the study only investigated patients in one metropolitan site and may not be generalisable to all subacute inpatient facilities or populations. Second the sample size of this study was smaller than ideal for a prevalence study which is reflected in the relatively broad confidence intervals for the prevalence rates in this study. However, via the confidence intervals reported in this study aligning with the prevalence results reported in other small studies [22,23], this study does potentially provide other similar subacute inpatient services with an evidence-based opportunity to forecast the foot disease prevalence ranges they may expect in their services. The small sample size also means that the study may have been underpowered to detect further significant associations for factors identified in the existing literature to cause multiple foot disease risk factors; for example diabetes and smoking. However, the study did have the sample size necessary to adequately test multivariate logistic regression models for up to four explanatory variables as performed in this study [27]. Lastly, a potential limitation was the reliability of data collection which was performed by a single podiatrist using the standard QHRFF. However, the QHRFF has been to shown to have the validity and reliability necessary to detect the variables included in this study [14]. Overall, to improve the generalisability of these results it would be strongly recommended that a similar multi-site study be performed with a population of over 300 participants as recommended when investigating prevalence of chronic wounds in particular [41].

To the best of the authors’ knowledge, this study does appear to be largest study, in terms of population numbers and response rates, to investigate active foot disease and foot disease risk factors in the subacute inpatient setting [22,23]. Furthermore, the primary aim of this study was to investigate the prevalence of clinically diagnosed foot disease complications and foot disease risk factors unlike other similar smaller studies [22,23]. In addition this study used what could be considered as gold standard non-invasive methods to clinically diagnose these foot disease risk factors and foot disease complications unlike other smaller studies [22,23]. In this period of rapid expansion in the subacute inpatient sector it is recommended that further multi-site studies are implemented to verify the generalisability of these findings and investigate the effect of foot disease on patient and service outcomes in these subacute inpatient facilities in particular. Furthermore interventions to reduce the foot disease burden as has happened in other at risk populations for foot disease should be investigated.

## Conclusion

This study provides timely foot disease and foot disease risk factor prevalence and associate data for future services and studies to utilise in this expanding subacute inpatient sector. Approximately one in eight patients had active foot disease, one in three had multiple foot disease risk factors and one in two had at least one foot disease risk factor, whilst admitted in this subacute inpatient rehabilitation facility. If these findings are generalisable to other subacute inpatient facilities then the burden of foot disease is comparable to other at risk populations for foot disease. This then poses the question whether similar management guidelines should also be implemented in subacute inpatient facilities as occurs in other at risk populations. These findings when viewed in the context of Australia’s ageing population and increasing burden of chronic disease suggests more has to be done to address the burden of foot disease in subacute inpatient rehabilitation facilities.

## Abbreviations

ANOVA: Analysis of variance
CKD: Chronic kidney disease
CVD: Cardiovascular disease
ESRF: End stage renal failure
OR: Odds ratio
PAD: Peripheral arterial disease
QHRFF: Queensland high risk foot form
SD: Standard deviation
UK: United Kingdom
